# Sharing fluids shapes host social networks and pathogen traits

**DOI:** 10.1098/rspb.2022.2493

**Published:** 2023-06-14

**Authors:** Jessica F. Stephenson

**Affiliations:** Department of Biological Sciences, University of Pittsburgh, Pittsburgh, PA 15260, USA

Host behaviour is a critical determinant of infectious disease dynamics: how individual animals interact with, or ‘contact’, each other dictates whether and which diseases transmit between them. For an example using pathogens of current concern among human populations, sharing an elevator with someone might lead to you contracting SARS-CoV-2 (which transmits through respiratory droplets), but it will not lead to you contracting monkeypox (which transmits through extended skin–skin contact) or HIV (which requires the exchange of blood, sexual fluids or breast milk). Animals should therefore evolve to be discriminatory about whom they contact, and those decisions may depend on the nature of that contact: indeed, you likely have different criteria for deciding with whom to share an elevator versus fluids. Similarly, pathogens should evolve traits that increase their transmission success, and these traits may differ depending on the type of contact required for transmission. These two ideas—that pathogens affect the evolution of host behaviour and host behaviour affects pathogen evolution—have long been assumed, but are notoriously difficult to test [1,2].

In a recent paper, Collier *et al*. [3] take an innovative, multi-pronged approach to testing how pathogen transmission may shape how individual animals interact with their groupmates, and how pathogens may evolve in response. First, the authors use a database of 232 published contact networks across six animal taxa—most collected to test questions unrelated to disease—to evaluate whether networks vulnerable to the transmission of similar pathogens share characteristics. The types of contacts used to build the networks vary substantially, and Collier *et al*. [3] categorize them from the perspective of a hypothetical pathogen: indirect contact (e.g. asynchronous space sharing), non-physical close contact (e.g. shared group membership), direct physical contact (e.g. allogrooming or fighting) and fluid exchange (e.g. penetrative sex). Thus categorized, the authors show that networks of fluid exchange differ significantly in many key metrics from the others, and in general tend to be sparser, more heterogeneous (i.e. some individuals contact many others, others few or none), and more fragmented.

Poor connectivity, as appears to characterize fluid exchange networks, should intuitively offer pathogens fewer transmission opportunities than the typically highly connected physical and non-physical contact networks, and thus select for different pathogen traits. Collier *et al*. [3] use an epidemiological model to evaluate the traits required for a hypothetical pathogen to persist on such networks and find that pathogens transmitting on fluid exchange networks must have significantly higher transmissibility than those using other contact types. To test this hypothesis, they collated data on human pathogens, for which transmissibility data are far better and more available than for wildlife pathogens. Two characteristics of transmissibility—infectious period and infection probability—revealed that those that transmit on fluid exchange networks tend to have the longest infectious periods, and those with shorter infectious periods compensate by having moderate to high infection probability.

Through this neat and logical series of steps, Collier *et al*. [3] show that, across a wide range of taxa, the structure of animal social networks is somewhat predictable by the nature of the contact on which they are built, and that this structure predicts pathogen traits. Of course, being largely meta-analytical in approach, Collier *et al*. [3] were constrained by data availability: many of these constraints represent important and exciting future research directions. Here I highlight a few outstanding examples.

The paper strongly suggests that the false dichotomy between diseases that transmit through sexual and non-sexual contact reduces our ability to understand transmission dynamics. Collier *et al*. [3] point out, as have others [4], that their categories of contact networks (except those built using indirect contacts) are nested: direct physical contact must be preceded by non-physical close contact, and, among non-human animals at least, fluid exchange (which, in their dataset is predominantly through sexual contact) must be preceded by physical contact (figure 1). Indeed, for diseases that *can* transmit through non-physical contact, it is likely that physical contact or contact involving fluid exchange will dramatically increase the chance of that transmission. Importantly, this point has recently been illustrated by monkeypox: while the virus does not require sexual contact to transmit, the extended skin–skin contact it does require is likely to happen during sex, and as a result the virus has, to date, mostly been infecting men who have sex with men [5]. Insights we have gained from studying sexually transmitted diseases thus likely apply to diseases not typically considered sexually transmitted, especially if their infection probability increases substantially with more intimate contact (i.e. moving towards the centre of figure 1).

**Figure 1.**
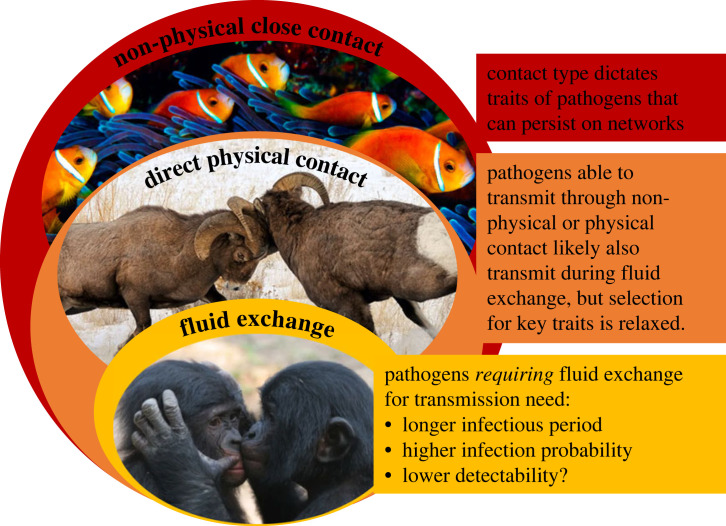
Pathogen transmission on host social networks should be considered a nested process. Collier *et al*. [3] categorize contacts between animals according to the transmission route they provide pathogens. Pathogens with airborne or respiratory droplet transmission can transmit between hosts during non-physical, close contact, such as occurs between members of a social group. Adding direct physical contact, such as fighting and allogrooming, additionally permits the transmission of pathogens that use skin-to-skin contact, but airborne or droplet transmitted pathogens can still spread on these networks. Similarly, when animals exchange saliva or other fluids, for example during sexual contact, an additional set of pathogens can travel, but skin-to-skin and respiratory transmitted pathogens can also still spread on these networks. Collier *et al*. [3] found support for this nested structure selecting for pathogen transmissibility, as explained in the text boxes, and I suggest it may also select against infection detectability. Adapted from figure S2 in Collier *et al*. [3]. Images: fish: David Doubilet/National Geographic; rams: Nancy Elwood/National Geographic; bonobos: theifod.com.

More broadly, sex is underrepresented in studies of wildlife disease ecology. For example, across taxa, tests of parasite-mediated sexual selection hypotheses have revealed that choosers can detect and avoid potential mates infected with parasites [6]. Of course, such individual-level behavioural decisions should affect whether and to whom parasites transmit, and there has been some theoretical consideration of how mate choice might affect the epidemiology of sexually transmitted diseases [7]. However, even though animals appear not to adjust this avoidance behaviour according to pathogen transmission route (e.g. avoiding potential mates infected with a parasite that poses no direct transmission risk [8]), there has been no consideration, to my knowledge, of the role of mate choice in the epidemiology of non-sexually transmitted diseases. This represents a potentially important oversight: in many species, an individual's non-physical and physical contacts, let alone fluid exchange contacts, will be directly related to the potential mates they are courting, regardless of whether that courtship is successful. While the interactions between infection and non-sexual social behaviour are already complex and require elucidation [1], sex is an important driver of animal behaviour, and the ways in which it affects pathogen transmission are underexplored. For example non-reproductive and, as Collier *et al*. [3] point out, same-sex sexual behaviour is hugely neglected in wildlife systems, but structures human social networks in ways important for predicting disease spread [9].

That individuals can detect and avoid infected conspecifics is an observation that Collier *et al*. [3] raise as a potential limitation of their study: they could not account for changes in network structure due to the presence of disease. The idea that host behavioural responses to the presence of disease may impact social networks and ongoing transmission is not new—indeed, it has long been a focus of theoretical studies in humans [10]. However, empirical data to validate these models are rare, even in human disease systems, and in general underscore the complexity of the interactions between infection and behaviour [11]. Overall, it remains unclear the extent to which ignoring this phenomenon might affect the conclusions Collier *et al*. [3] draw from their non-human animal social networks. On one hand, we know that the presence of infection can affect the behaviour of both infected and uninfected hosts, but these effects are highly nuanced [1]. For example, uninfected hosts may adjust their behaviour based on their own susceptibility to infection [12–14] and the risk that the infected host will transmit the infection [15]. The effects of infection on infected host behaviour also likely depend on the intensity of that infection [12], and the individual host's tolerance [16], both of which can vary substantially between individuals and through time. Predicting how the structure of host social networks will change during an outbreak is therefore challenging, particularly as we have almost no empirical data from non-human systems (but see [17,18]).

To further complicate this picture, how the presence of a disease affects network structure likely depends on which of the four contact types identified by Collier *et al*. [3] the pathogen uses for transmission. Pathological effects of infection may well change how and with whom infected hosts interact [19], though these effects can sometimes be supressed in contexts where their costs are too great [20,21]. For uninfected hosts to avoid diseased conspecifics, they must both perceive the disease and decide that the appropriate response is avoidance. The feasibility of each of these steps depends on the symptoms of the disease, and the costs associated with avoiding contact with conspecifics. Obvious symptoms associated with obvious disease in a conspecific provide excellent cues for avoidance, but the more intimate the contact required for transmission, the stronger the selection on pathogens to conceal such cues. Perhaps as a consequence of this selection, across host and parasite taxa sexually transmitted diseases tend to be less obvious than ordinary infectious diseases: they are more asymptomatic, with more localized symptoms, and less central nervous system involvement [22].

Further, while the costs of missing out on each of the contact types in figure 1 are likely highly system and context dependent, they may well constrain changes in network structure. For example, non-physical contact is a key component of anti-predator defence for many animals [23], so these networks may be particularly constrained. However, infection-induced pathology does appear to affect host ability to maintain anti-predator behaviour across systems [24,25] and can change interactions among prey under the threat of predation [26]. The cost of lost mating opportunities either directly or through changes in intrasexual competition may also constrain changes in direct contact and fluid exchange networks. Some support for the idea that the costs of avoiding mating constrain changes in animal behaviour comes from the observation that males may show little, or no avoidance of even very obviously diseased females, whereas females are more likely to avoid mating with infected males ([27] and references therein). This sex difference is in line with classic parental investment theory [28]: in general, in iteroparous species with typical sex roles, females require post-mating health to maximize offspring quality and the number of successive broods they can produce, whereas males can sire many more broods during a single mating season. The substantial variation across systems in their reproductive ecology represents an exciting opportunity to test how the value of present versus future mating opportunities may or may not constrain how infection affects fluid exchange networks. In summary, although Collier *et al*. [3] are correct to raise infection-induced behavioural change as a potential limitation of their study, their overall inferences may be fairly robust: in many systems, hosts may not perceive the pathogens that transmit through such networks, or not associate their cues with disease, and thus not adjust their behaviour, or the costs of doing so may be too great.

Overall, Collier *et al*. [3] link three pieces of evidence to show how parasite transmission may have shaped the structure of host social networks, and how those structures may have in turn shaped the evolution of pathogen traits. The paper is a thoughtful and comprehensive use of the data available, and hopefully its constraints will serve as motivation for many more. In particular, the nested structure of the types of contacts pathogens use for transmission highlights how research on sexually and non-sexually transmitted diseases may benefit from considering such diseases on a continuum that better reflects host contact behaviour. This shift could usefully accompany more comprehensive study of sexual behaviour in non-human animals: sex in all its forms has been historically understudied in non-human animals but is an important driver of animal behaviour and thus pathogen transmission. Finally, while we have some evidence that network structures may change as epidemics spread [17,18], it remains unknown how that rewiring may depend on, and affect the evolution of, parasite transmission route or other traits such as detectability.

## Data Availability

This article has no data.
